# Toward Self-Referential Autonomous Learning of Object and Situation Models

**DOI:** 10.1007/s12559-016-9407-7

**Published:** 2016-04-27

**Authors:** Florian Damerow, Andreas Knoblauch, Ursula Körner, Julian Eggert, Edgar Körner

**Affiliations:** 1 Control Theory and Robotics, Technical University of Darmstadt, 64283 Darmstadt, Germany; 2 Faculty of Computer Science, Albstadt-Sigmaringen University, 72458 Albstadt, Germany; 3 Honda Research Institute Europe GmbH, Carl-Legien-Straße 30, 63073 Offenbach, Germany

**Keywords:** Self-referential control, Scene understanding, Autonomous learning, Hierarchical situation model

## Abstract

Most current approaches to scene understanding lack the capability to adapt object and situation models to behavioral needs not anticipated by the human system designer. Here, we give a detailed description of a system architecture for self-referential autonomous learning which enables the refinement of object and situation models during operation in order to optimize behavior. This includes structural learning of hierarchical models for situations and behaviors that is triggered by a mismatch between expected and actual action outcome. Besides proposing architectural concepts, we also describe a first implementation of our system within a simulated traffic scenario to demonstrate the feasibility of our approach.

## Introduction

Scene understanding and situation recognition is an important prerequisite for realizing intelligent mobile support systems for humans, for example, driver assistance systems or systems for autonomous driving [1–4]. Current driver assistance systems provide comfort functions such as lane keeping, pedestrian recognition, and cruise control which have reasonably good performance because sensory analysis is restricted to the recognition of a small set of simple predefined object and situation templates (e.g., [5–7]). However, when trying to perform the step from comfort functions toward autonomous behavior, the situation complexity reaches a level, where hand constructed situation templates will fail, because they lack the capability to adapt to behavioral needs not anticipated by the human modeler of the template. For example, many current approaches used in computer vision for situation understanding are mostly based on low-level sensory representation which are by principle not able to acquire relevant information for behavior adaption [8–12] Other approaches try a “full” semantic representation of the scene including the recognition of all visible objects and situations for which a template is available [13–15]. Such approaches soon arrive at hard computational limits and generally do not scale to real-world applications, because they neglect to focus analysis on the most relevant items that are necessary for making autonomous decisions and reaching behavioral goals.

Thus, current systems seem to lack the capability to adapt object and situation models adequately to the actual behavioral needs defined by the situational context the system is embodied in. To approach this problem, we have worked out in the last years a brain-inspired cognitive architecture for “self-referential” autonomous learning of hierarchical knowledge representations [16–19]. Key elements of our architecture are the acquisition of knowledge based on behavioral needs and the reusability of parts of the already acquired knowledge to explain new situations. More specifically, acquisition or adaptation of object and situation models is triggered by a mismatch between actual behavioral outcome derived from the current sensory input signals, and the expected outcome derived from previously learned internal world models (cf., [20]). Although this process is computationally expensive, our system is able to at least partly automatize the process of creating and adapting models for objects, situations and behavior.

In this work, we give a detailed description of our current implementation of the system architecture for self-referential autonomous learning, which enables the refinement of already-gathered knowledge by new experience to improve the system’s behavior. For example, our architecture may enable a human designer to define the system’s task by hand-engineering a corresponding high-level behavioral model that includes situations and their corresponding sensory representations, expected outcomes of relevant actions, and a reward function on the situation states to specify the goal of the task. Although this hand-designed model is easily integrated into the system’s concept ontology by making references to already existing concepts, it will typically be incomplete because the human designer will not be able to identify all relevant model components for solving the task (not to mention model parameters). Our system enables now the refinement of the existing knowledge representations by refining the object and situation models in parallel with the agent’s behavior. That is, starting from a low-dimensional imprecise state space, the mechanism of self-referential autonomous learning expands the situation space according to behavioral needs. Due to the hierarchical knowledge organization, an iterative refinement of both situation and behavioral models is possible. Our work demonstrates how such a process can quickly improve system performance.

The paper is organized as follows: Section 2 introduces the basic concepts including the format of hierarchical situation and action models, and a description of structural learning for specializing or generalizing these models. Then Section 3 describes the current implementation of our architecture within the context of a simple simulated traffic scenario, where a car agent learns to drive safely over a zebra crossing. Section 4 shows corresponding simulation results. Finally, Section 5 concludes and discusses our approach in the context of possible applications and alternative approaches.

## Basic Concepts

### Scenes versus Situations

It is important to distinguish between the two terms “scene” and “situation”: We define a *scene* as a set of entities that give a rich, potentially complete, perceptual description of the current sensory environment of the subject. By contrast, we define a *situation* as a behaviorally relevant state of a subject, closely related to action options and outcomes.

We will give more precise definitions below, but want to emphasize here that, according to our definitions, a scene refers predominantly to the perceivable environment describing the circumfluent space. In contrast, a situation is rather the task-driven interpretation of a scene referring also to behavioral models, action outcomes, and internal states of the subject such as intentions or goals. In this view, a situation model mediates between perception and action by defining behavioral relevant scene entities necessary to recognize the situation as well as providing necessary parameters for associated actions. While scenes are potentially full descriptions of the perceptually sensible entities in the external environment, situations include only behaviorally relevant entities that are necessary to recognize the situation and/or to initiate appropriate actions.

Such a distinction between “scene” and “situation” is largely consistent with the etymologies of the two terms and is as well reflected in brain structure. In fact, the brain has separate centers for realizing a sensory “scene” memory in the retrosplenial and in the parahippocampal cortex integrating currently processed objects within the current spatial context [21–23], and another set of structures for realizing a “behavioral” working memory in the frontal cortex and associated regions that integrate sensory entities with the current situational context including current goals, action options, and expected outcomes [24–26].

Note that our idea of a situation differs from situation calculus [27] as we cannot easily identify a situation with a sequence of actions or a universal state. Unlike in common Markov decision processes (MDPs) [28, 29] or partially observable MDPs [30, 31], our idea of situations corresponds to neither a fixed set of states nor observations, but rather assumes a more flexible dynamic structure as we will see below in more detail. This includes, for example, hierarchical organization and, unlike many hierarchical MDP approaches [32], also learning mechanisms for re-structuring by specialization and generalization.

### Knowledge Representation

#### Hierarchy of Scene Entity Models

By scene entities, we denote elements of a scene such as objects, situations, relations between other scene entities, and spatial layouts being containers for other scene entities. Each scene entity model specifies the process of sensory recognition of the scene entity, for example, by defining type and locations of relevant parts to attend to. All scene entity models are included as nodes in a graphical structure that we call hierarchical scene entity model. Basically, the hierarchical scene entity model contains a has-parts ontology and an is-a ontology. The former describes the decomposition of a higher-level concept into several lower-level parts, and the latter covers variances by allowing several subtypes of a concept.

Our model has close relationships to previously proposed standard models for brain-inspired object recognition. For example, biological neural network models often consist of a hierarchical arrangement of simple (S) and complex (C) cells that employs similar mechanisms as our model to represent part-whole (S) and type-subtype-relationships (C) (e.g., [10, 11]): S cells essentially implement an AND operation, i.e., an S cell gets activated if there is sensory evidence for part 1 AND part 2 AND part 3. Similarly, C cells implement an OR operation, i.e., a C cells gets activated if there is sensory evidence for configuration 1 OR configuration 2 OR configuration 3. Within a probabilistic framework, one can think of such models as being composed of AND and OR layers resulting in a polytree-like graphical structure without any loops for which there exist efficient belief propagation methods such as the sum-product and max-sum algorithms (e.g., see [33]).

**Fig. 1:**
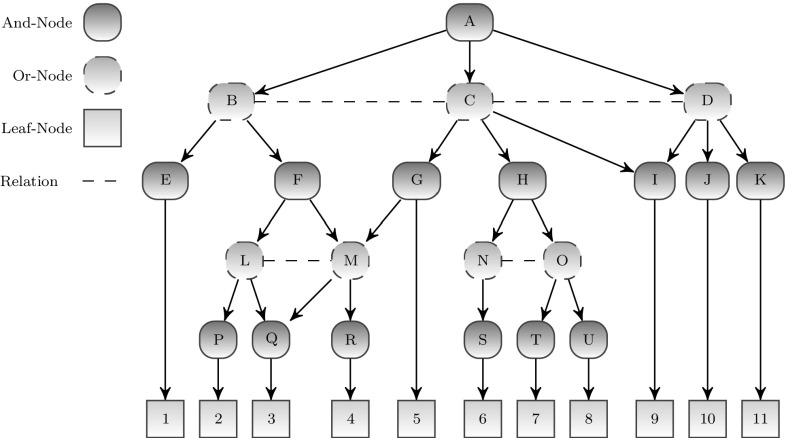
And-Or-Graph by Zhu [13]

Figure 1 illustrates a related model by Zhu and Mumford [13]. This so-called AND/OR graph (AOG) model is again a hierarchy of AND and OR node layers within a probabilistic framework as discussed above. However, it extends the tree-type standard models by horizontal links within the OR layers to express relations between the parts of an AND node. Note that such links introduce loops such that exact probabilistic inference becomes infeasible in general.

**Fig. 2:**
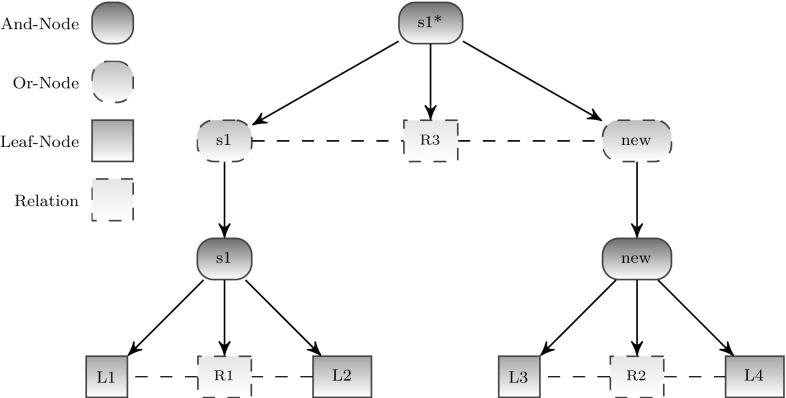
Hierarchical scene entity model

At the present stage of research, the hierarchical scene entity model is represented as a deterministic AND-OR-graph as illustrated in Fig. 2 and closely related to [13, 34]. Here, a relation corresponds to a special node below an AND node. To check if a certain situation represented by an AND node holds, first, all non-relation children of the AND node have to be checked, before it can be determined whether the children are in a certain relation. Each of the AND and OR nodes may represent the sensory configuration for recognizing a certain situation.

As shown in Fig. 2, a specialization $s_1^*$ of a situation $s_1$ is located above the layer of $s_1$. The refined situation $s_1^*$ adds more details (node ${ new}$) to the sensory configuration of $s_1$. Thus, a consecutive refinement process of the sensory configuration is reached by going upwards into the specializations of a certain situation.

#### Situation Model

Formally, we define a situation model as a triple $s=({ sc}, F, A)$. The first component ${ sc}$ is a link to a scene entity model as explained in the previous section. This link basically defines how to test whether situation *s* holds by analyzing the sensory input.

Second, $$F=\{f_1,f_2,\ldots \}$$ is a set of expected feedbacks $$f_i=(l,r)$$ that may occur in situation *s*, where *l* is a textual label and *r* is a real-valued expected reward. Several expected feedbacks are possible in one situation. For example the situation $$s_1$$ “drive safely over zebra crossing” contains further sub-situations such as $$s_{11}$$ “stopped at zebra crossing” and $$s_{12}$$ “crossed zebra crossing.” Those sub-situations expect different feedbacks $$F_{11}$$ and $$F_{12}$$, respectively, with labels $$f_{11} = ('{} { reward}\, { for}\, { stopping}\, { correctly}\, { at}\, z.c.',0.1)$$ and $$f_{12} = ('{} { reward for safely passing} z.c.',2.0)$$. Correspondingly, the higher-level situation $$s_1=$$“drive safely over zebra crossing” then expects both feedback types with $$F_1 = \{f_{11},f_{12}\}$$.

Finally, $A=\{a_1,a_2,\ldots \}$ is a set of possible actions $a_i$ that may be executed in situation *s*. The following section gives a formal description of actions.

#### Action Model

In each situation $s_i$ a set of actions

$$a_j= (s_{{init}}, bm_{{low}}, s_{{goal}}, a_{{act}})$$

can be performed which may result in a situation transition. Formally, we define an action as a quadruple $a=(s_{{init}},{ bm}_{{low}},s_{{goal}},a_{{act}})$, where we distinguish two types: First, a low-level action $a_{{low}}=(s_{{init}}=None, bm_{{low}}={None}, s_{{goal}}={None}, a_{{act}}\ne {None})$ controls directly relevant actuator states such as the gas pedal position of a car by performing the actuator function $a_{{act}}$ which is a parametrized interface to the actual actuators of the agent. Second, a high-level action $a_{{high}}=(s_{{init}}\ne {None},{bm}_{{low}}\ne {None},s_{{goal}}\ne {None}, a_{{act}}={None})$ consists of (1) a (higher-level) initial situation $s_{{init}}$ which defines the execution condition for the action, (2) a behavioral model ${bm}_{{low}}$ defining a (lower-level) policy to execute the action, and (3) a (higher-level) goal situation $s_{{goal}}$ which defines the deactivation condition of the action. Thus, actions have a hierarchical structure via behavioral models that comprise a detailed (lower-level) plan to execute the (higher-level) action and reach the desired goal. This is explained in more detail by the following section.

**Fig. 3:**
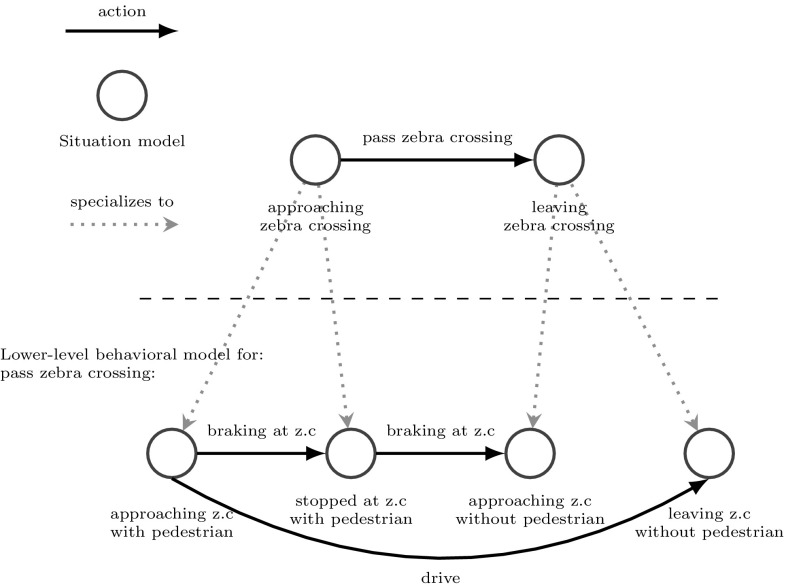
*Hierarchical organization of behavioral models.* In this example, executing the action “pass zebra crossing” activates the lower-level behavioral model, which itself executes different low-level actions. The* green arrows* represent the specialization of situation models (Color figure online)

#### Hierarchical Behavioral Model

Situations are always embedded in behavior. This means that each situation $s_i$ affords a set of actions $a_i$ that could be performed. Each action will lead to a situation transition $s_{t+1}=f(s_t,a_t)$. Such transitions can be represented as a graph or, more generally, as a Markov model with transition probabilities $pr[s_{t+1}|s_t,a_t]$. Thus, a behavioral model is closely related to the theory of Markov decision processes (MPD) [35] and can be modeled as ${bm}=(S,A,P,R)$ where *S* is a set of situation models, *A* is a set of actions, $P=S \times A \times S \rightarrow [0, 1]$ is the transition distribution, and $R(s_{t+1}|s_t,a_t)$ is the expected reward. Fig. 3 illustrates a behavioral model as a graph where nodes denote situations and arrows correspond to lower-level actions intending certain situation transitions. Note that behavioral models have a hierarchical structure because each lower-level action may consist again of a lower-level behavioral model.

A situation node *s* in a behavioral model *bm* executed through a higher-level action $a_{{high}}$ can be of the following types:

- Initial Situation $$s_{{init}}$$
  An initial situation of ${ bm}$ is a specialization of the initial situation of $a_{{high}}$ in the corresponding higher-level behavioral model.
- Goal Situation $$s_{{goal}}$$
  A goal situation of ${ bm}$ is a specialization of the goal situation $a_{high}$ in the corresponding higher-level behavioral model.
- Else Situation $$s_{{else}}$$
  This situation is active if no other situation is active. Only one situation of this type is possible in one behavioral model.

The set of all initial situations may also be called the *initiation set*, and the set of goal situations the *termination set* of an action. Our idea of actions is thus similar to *options* as employed in hierarchical reinforcement learning models [32, 36], although, due to the self-referential learning of situation and behavioral models, we cannot distinguish as sharply between primitive actions and options or, correspondingly, between the lowest level “core” MDP and option policies.

**Fig. 4:**
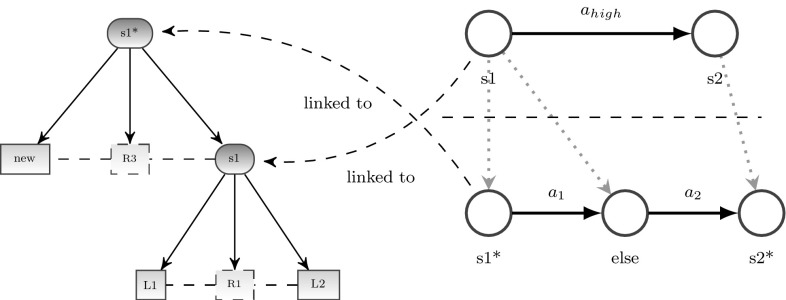
Links between scene entity model (*left*) and behavioral models (*right*). Behavioral models are strongly connected to the scene entity model. Iteratively refining the behavioral model means in parallel refining the scene entity model

It can be seen in Fig. 4 that descending in the behavioral model corresponds to ascending in the sensory configuration in order to gain a more detailed situation description for a refined behavioral performance. For example, if the situation $s_1$ holds, the associated high-level action $a_{{high}}$ is performed, which means activating a lower-level behavioral model. The initial situation of this behavioral model is a specialization $s_1^*$ of $s_1$. To check if $s_1^*$ holds only the new part of the graph has to be checked as $s_1$ had already been checked before. This enables a fast analysis of the current situation, because first a quite general situation, which can be evaluated very quickly, is checked before refinement starts iteratively. In case the refinement process has to be truncated (e.g., because of a time-out), an approximation of a situation is already available, which might be sufficient to change behavior toward reaching a goal. To check if a certain situation holds, the situation model checks the sensory configuration by testing if all conditions for this situation hold. This means that the subgraph of the concerning situation node has to be parsed. Parsing the situation subgraph includes checking if leaf node objects are present and relations hold. At this stage of research, the parse algorithm is deterministic. But as our parse algorithm is based on [34], it is possible to extend it to probabilistic inference.

A simple control strategy for the efficient execution of such hierarchical behavioral models is to realize a competition between relevant actions at each level of representation that is based on evaluating activation/deactivation conditions and expected rewards:

1. Activation condition. An action can be performed if the activation condition holds, i.e., if the subject is in a situation that allows performing the action.
2. Deactivation condition. The action will be deactivated if the deactivation condition holds, i.e., if the subject is in an appropriate end situation.

For example, a high-level action representation would be activated only if the initial condition holds and the action is associated with the highest expected reward among all possible actions. Similarly, a lower-level action would be activated only if requested by the higher-level action, i.e., if it is part of the behavioral model of the active high-level action.

As discussed in further sections, it is the major target of self-referential control to learn and/or extend such semantic ontologies of situation and behavioral models in an autonomous goal-directed way.

### Self-Referential Control of Autonomous Learning

So far we have described concepts and structures for knowledge representation such as situations, actions, and behavioral models. Although they may be hard-coded by a designer, here we are particularly interested in autonomously learning these structures. As learning from scratch is difficult to demonstrate, we rather assume that a rich set of structures is already provided and discuss how to extend the system by “self-referential“ autonomous learning. Here the qualification “self-referential” (coined by Körner [37]) refers to the fact that the system can interpret the environment only in terms of “its own” previously acquired situation models and has to integrate previous and novel models in a consistent behavior-related way.

In this work, we focus on teacher-guided “top-down” learning: We assume that an external “teacher“ communicates an abstract high-level behavioral model for solving a particular task that typically refers only to high-level situations and actions. In the subsequent learning phase, the system has then to refine the teacher model based on its own behavioral experience. Unlike in unconstrained “bottom-up” learning, the teacher model, although symbolic and ungrounded, can strongly reduce the search space of possible actions and guide the system quickly toward the desired behavior. Such a process is similar to learning in humans, as most “real“ human behavioral models have not been acquired by pure exploration, but have rather been transmitted in a cultural process from teacher to student.

So we can assume that there is already a behavioral model for a higher-level action, and the system may try to behave accordingly in order to reach the goal of that action. As the teacher can communicate only a symbolic behavioral model, the system still has to ground the model operationally. This means that the system collects episodic data by trying to perform the actions suggested by the teacher model until it arrives in the desired goal state. During subsequent learning, the episodic data are used to ”complete” the situation and action models provided by the teacher and integrate them into the hierarchy of scene entity and behavioral models. In general, this will involve several distinct learning processes including

1. Structural learning and refinement of the behavioral models,
2. Learning of corresponding feature representations in order to be able to classify situations correctly based on sensory inputs, and
3. Learning parameters and values of preferred actions.

The latter two learning problems are relatively well established, at least if considered in isolation, and assuming a fixed situation space. Problem 3 is usually solved in a control approach, in our work by the Q-learning algorithm which is a specific reinforcement learning (RL) algorithm [29]. The algorithm has a function $Q_i:S_i \times A_i \rightarrow R$ which calculates the quality of performing action $a_{{in}}$ in situation $s_{{im}}$ for each behavioral model ${ bm}_i$. Problem 2 is usually solved by standard supervised or unsupervised pattern recognition and machine-learning approaches. We employ an algorithm for information generation as explained further below to generate the learning data necessary to extend and refine the sensory scene representation. For the structural learning problem 1, we suggest a novel approach where extending the model structure is based on the detection of certain graph motifs in behavioral models. This approach includes the structural learning concepts of specialization and generalization which are explained in the following.

#### Specialization

It has been proposed that the brain uses the difference between expected and actual sensory experience as a trigger for knowledge generation [38]. Based on this principle of knowledge generation, we introduce a mechanism for structural learning to refine the existing knowledge base.

**Fig. 5:**
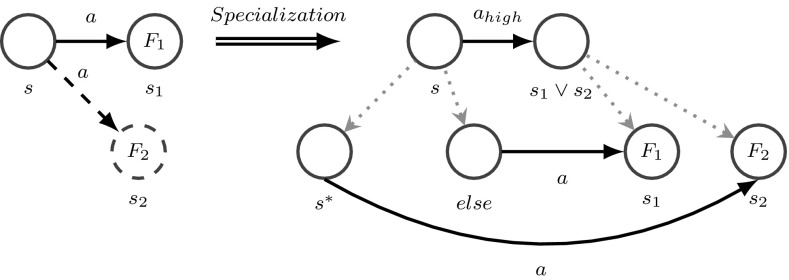
Specialization refines the behavioral model as well as the scene entity model to improve the behavior

The trigger for specialization is the graph motif illustrated in Fig. 5 (left) which shows a clip from a behavioral model where performing action *a* in situation *s* may lead either to the expected situation $s_1$ with the expected feedback $F_1$ or to the unexpected situation $s_2$ with an unexpected feedback $F_2$. Such a motif expresses basically the uncertainty of the system as it is unable to predict the outcome of a certain action in a certain situation. In our system such a graph motif triggers structural learning in order to reduce uncertainty and increase the system’s ability to predict future situations. Here, this means to extend the behavioral model, especially replacing action *a* by a high-level action $a_{{high}}$ through specializing situation *s* into two new situations $s^*$ and $s_{{else}}$ such that executing action *a* in situation $s_{{ else}}$ leads to the previously expected situation $s_1$, whereas choosing *a* in situation $s^*$ leads to the new situation $s_2$ that was unexpected previously. Thus, the refined behavioral model can better predict the outcome of performing action *a* and react accordingly.

Once the specialization process is completed, for every new situation the optimal action can be determined via reinforcement learning. This learning of the optimal policy can use already experienced memory to pre-learn from mind. This allows to make a more accurate educated guess for the optimal action in the next learning cycle.

Although here we implement only on a simple deterministic simulation scenario, realistic stochastic environments imply that certain situation transitions may depend on factors not observable by the agent. In that case, the described specialization procedure would not be able to improve the agent’s performance. To avoid uncontrolled proliferation of specialized situation models, it would then be necessary to extend our model with methods that can deal with partially observable or hidden factors [31, 39]. Another mechanism counteracting specialization is generalization as described in the following section.

#### Generalization

**Fig. 6:**
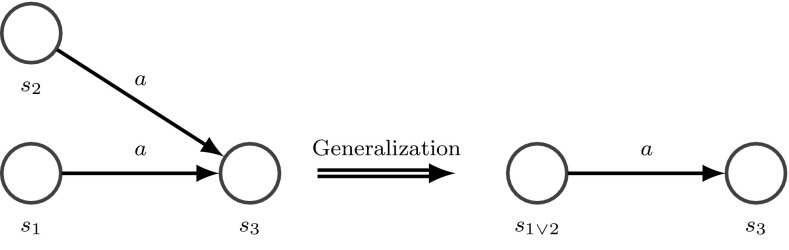
Generalization combines situations having the same optimal action that lead to the same successor situation. Thus, generalization avoids an unnecessary separation of situations that have a similar action/outcome relation

Another graph motif that triggers structural learning is shown in Fig. 6, left. Here, the system has learned a behavioral model where an outcome $s_3$ can be reached from two different initial situations $s_1$ and $s_2$ by executing the same action *a*. Such a graph motif means that situations $s_1$ and $s_2$ are similar because both can lead to $s_3$ by executing *a*. Therefore, it makes sense to generalize both involved situations $s_1$ and $s_2$ to a novel situation model $s_{1 \vee 2}$ where action *a* can be performed (Fig. 6, right). Such structural generalization renders a more compact description of the fact that we can reach $s_3$ from $s_{1 \vee 2}$ by choosing *a*. This can potentially simplify behavioral models and accelerate prediction and planning. In the simplest case, the scene entity model of $s_{1 \vee 2}$ is an OR connection of the sensory configurations of $s_1$ and $s_2$. By applying graph reduction tools this newly connected graph can be further reduced. Thus generalization is used to compress unnecessary specialized situations or acquire novel higher-level symbols [40]. A situation is unnecessarily specialized if the optimal action determined via policy learning is identical to the optimal actions in the specializations.

## Implementation

We have realized the self-referential (SR) learning architecture within a reinforcement learning framework. For our simulation experiments, we have employed the microscopic traffic simulator CarD [41]. The following describes the implementation of an agent that is capable of SR learning.

### Simplifications for Sensory and Scene Representation

As the focus of the current work is on the basic mechanisms of self-referential learning rather than low-level sensory representation and object classification, we have employed the following simplifications:

1. Ideal Object Detector
  We assume an ideal object detector, which is able to deliver all visible objects in the scene as well as corresponding object attributes without any uncertainty. This simplifies semantic information generation and reduces full probabilistic inference at the object level to a simple deterministic framework. At a later stage of research, we will include also uncertainty and attention control.
2. Object Oriented Scene
  The entire scene can be represented as a set *O* of objects $o_i=({ At})$. Each object has a set ${ At}$ of attributes ${ at}_i=({ atl}_i, { atv}_i)$, where ${ al}_i$ is an attribute label and ${ atv}_i$ the attribute value. Every object contains the integer-valued attribute with ${ atl}_i=$’${ category}$,’ which defines the category of an object (e.g., ${ at}_i=('{} { category}', 1)$, where 1 defines the category for cars). Such a representation vastly simplifies and speeds up recognition tasks because higher-level processes do not have to cope with lower-level feature and object detection problems.

### Self-Referential Learning Architecture

Similar to common RL frameworks, the agent has to perform certain tasks in a behavioral environment associated with certain rewards. The SR agent contains the self-referential autonomous learning architecture as introduced before. The agent is able to sense the world only through certain sensors, but is not aware of the entire world state. In the current implementation, the sensor is an ideal object detector that detects objects in a certain range in front of the car. As explained above, the detector returns for each object certain attributes including category, position, orientation, and speed.

**Fig. 7:**
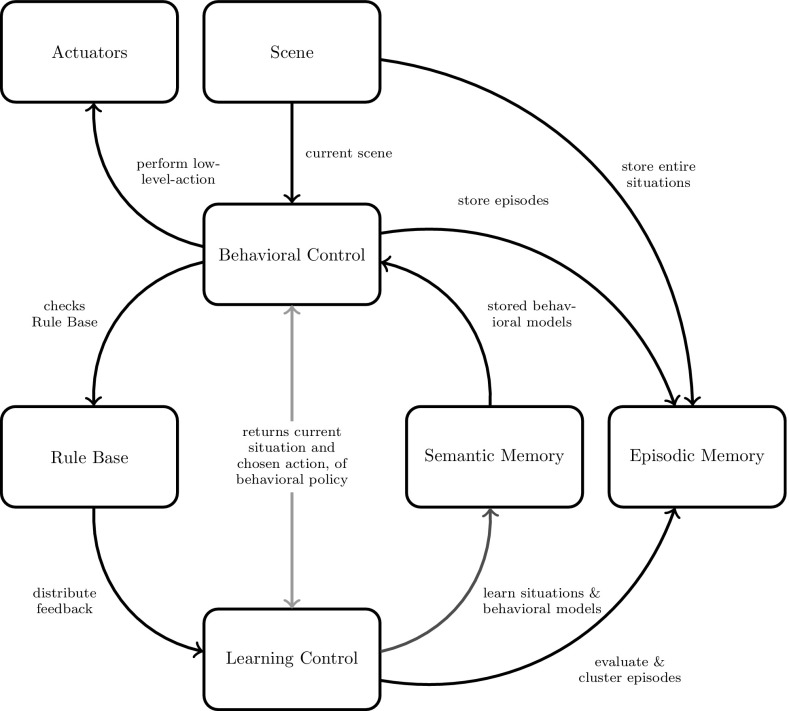
Self-referential learning framework determines the interactions between semantic memory, episodic memory, learning control, and the environment. *Red arrow* corresponds to self-referential learning (Color figure online)

At this stage of research, the agent’s actions are limited to longitudinal control, that is, setting gas and brake pedal pressure of the car. For simplicity we have not yet included transversal control like steering. Another input to the agent is a feedback signal $f=(l, r)$ comprising a label *l* and an actual reward *r* that enable the agent to evaluate and improve behavioral and recognition performance. The interaction of all important elements of the learning framework is illustrated in Fig. 7.

To enable SR control of learning as introduced by Körner [1], the agent needs a semantic memory, an episodic memory and some kind of learning control which will be introduced in the following.

For SR learning it is insufficient to store only those entities that have already a representation in the situation models of semantic memory. Rather, it is also necessary to store additional details of experienced behavioral episodes in order to discover new relations between sensory input and action outcome and learn new situation and behavioral models. For that purpose, we introduce two-memory systems: First, a semantic memory system for situation and behavioral models that represent only behaviorally relevant knowledge extracted from many behavioral episodes. And, second, an episodic memory to store detailed representations of individual behavioral episodes. As explained below, such episodes are linked to the semantic models to give a concise interpretation of what has happened, but they comprise also additional details to enable learning of new knowledge representations.

In correspondence to the two memory systems, we need two control structures for behavior and learning: First, the behavioral control unit employs behavioral models of semantic memory to generate optimal behavior based on recognized situations and calculated action values. Second, based on unexpected behavioral episodes, the learning control unit triggers certain learning mechanisms to generate refined semantic models for situations and behavior.

### Semantic Memory

As explained above, the semantic memory stores plans, how to execute actions according to situations as well as scene entity models for the recognition of situations. The semantic memory contains all the knowledge collected and understood by the agent.

The semantic memory can be modeled as ${ SM}=({ BM}, { SC})$, where *BM* is a set of hierarchical behavioral models ${ bm}_i$ and ${ SC}$ a set of hierarchical scene entity models. Each situation $s_j$ in each ${ bm}_i$ is connected to a node ${ (sc)}_j$ in the scene entity model. A situation can only be determined through the combination of its behavioral context, given by the location in a behavioral model and the scene entity model.

### Episodic Memory

An essential part of the SR framework is the episodic memory. The episodic memory is necessary to generate new semantic knowledge. More exactly, it is necessary to store episodic data that cannot be explained by the current semantic models (and that may indeed turn out to be irrelevant for behavior) in order to be able to refine the semantic knowledge base at a later time when a sufficient amount of episodic data has been collected. Such episodes must be stored in a well structured format to allow the agent to link the unexplained episodic data to the relevant behavioral context. Only then the agent will be able to extract relevant aspects and include them in the correct situation and behavioral models of semantic memory. This means that episodic memories must have a similar hierarchical order as behavioral models. Therefore, we have modeled hierarchical episodic memory as ${ EM}=({ ES}, { EP})$, where ${ ES}$ is a set of entire situations ${ es}$ and ${ EP}$ is a set of episodes ${ ep}$.

*An entire situation* is basically a snapshot of the whole scene at time *t* and can be modeled as ${ es}_t=({ SO}_t, F_t, { AC}_t)$, where ${ SO}$ is a set of Objects *O* sensed at time *t*, *F* the gathered feedback and ${ AC}$ the action control at time *t*. ${ AC}$ represents the current state of a behavioral model at time *t*, specifically the performed hierarchical actions as well as the active hierarchical situation models. It contains all visible objects with its attributes, a hierarchical snapshot of the active actions and situations and the received feedback. Before storing the received feedback, it is checked against the expected feedback by the agent’s world model.

**Fig. 8:**
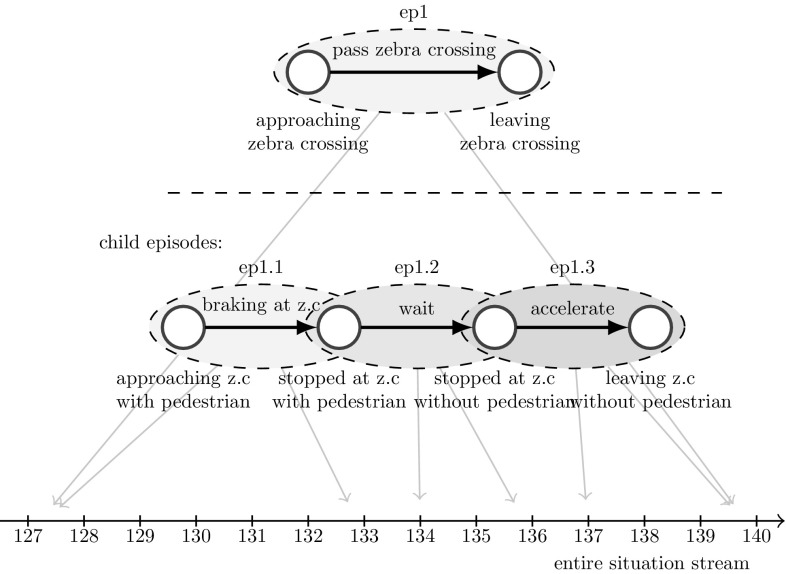
Hierarchical episodic memory orders the stream of entire situations according to the hierarchy of the executed behavioral model

*An episode* structures the stream of entire situations according to the behavioral model that has been executed by the behavioral control during experiencing the episode. Thus, an episode may contain several lower-level episodes reflecting the hierarchical structure of the corresponding behavioral model as illustrated in Fig. 8. This allows the agent to address entire situations in relation to corresponding “start situation - action - end situation transitions”: An episode can be modeled as ${ ep} = (s_{{start}}, s_{{end}}, a, (t(s_{{start}}), t(s_{{end}})))$ and is defined as the transition from a start situation $s_{{start}}$ to an end situation $s_{{end}}$ by performing the action *a*. $t(s_{{start}})$ is the time index of the first occurrence of the start situation and $t(s_{{end}})$ the first occurrence of the end situation. Thus, all entire situations ${ es}_{(t(s_{{start}})\le t \le t(s_{{end}}))}$ belong to the episode ${ ep}$. As mentioned before, an action *a* may be either a low-level action or a high-level action containing a lower-level behavioral model. Thus, episodes are structured according to the same hierarchical order as behavioral models.

The hierarchical structure of the episodic memory is illustrated in Fig. 8.

### Behavioral Control

The behavioral control ${ BC}$ is the control unit to execute the behavioral model. This means, first, to check whether certain situations are active (by employing the corresponding scene entity models) and, second, to choose an optimal action by evaluating action values of all possible actions. For each active behavioral model, a separate behavioral control unit is active. Due to the hierarchical structure of behavioral models, there are usually several behavioral control units active at the same time, which check situations and select actions on different levels of abstraction, down to the lowest and finest behavioral model that executes a low-level action. Here, any higher-level action executes recursively lower-level behavioral models and, thus, activates the corresponding behavioral control units on each level.

### Learning Control

The learning control unit ${ LC}$ is the basic control instance for any learning task. For example, as mentioned before, the mechanism of specialization learning is triggered by the graph motif where starting from one situation *s*1 and performing action *a* can lead to two different situations, one expected and the other unexpected. So the trigger for learning is the difference between sensory expectation and experience.

In this first proposal of an implementation, we use the feedback $f=(l, r)$ as a measure of expectation. Thus, any differences in expected feedback and actually received feedback activates structural learning. A fully trained behavioral model is then able to explain (meaning receiving a certain feedback in a situation that expects receiving this feedback) and predict (meaning that the resulting feedback of a certain action in a certain situation is known) feedback.

**Fig. 9:**
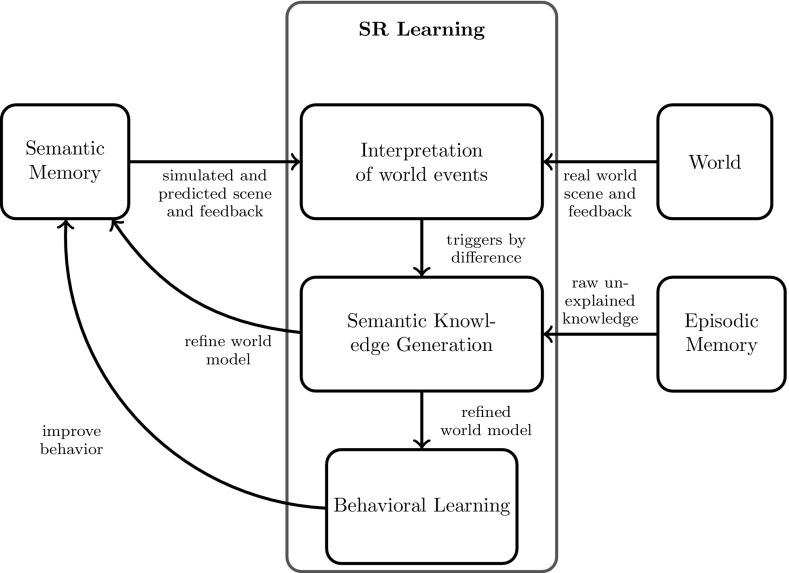
Self-referential learning. Difference between expected feedback from the internal world model and the real-world feedback triggers the semantic knowledge generation to refine the internal world model, followed by the behavioral learning to optimize the behavior

The overall learning architecture is illustrated in Fig. 9 and is divided into three sub-units dealing with interpretation of world events, semantic knowledge generation, and behavioral learning.

- Interpretation of world events
  Compares the sensory input with the internal world model. A difference in expectation and real-world experience triggers semantic knowledge generation. In our implementation, this means that, at every timestep, the received feedback is compared to the feedback expected in the active situation at every active level of abstraction. Such gathered feedback *F* is then included together with the expected feedback in each entire situation that is stored in the episodic memory. By this it becomes possible to extract information about which situation–action combinations lead to unexpected successor situations to control structural learning described below. Although potentially large amounts of episodic data are gathered at every time step, offline adaptation of situation and behavior models remains feasible because actual rewards $r>0$ are sparse events that occur typically only if an action has been successfully completed (i.e., if a higher-level goal state has been reached).
- Semantic knowledge generation
  A continued difference in expectation indicates that the actual situation (state) space is not sufficient to perform a given situation transition by a certain action. Thus, the situation (or state)-space has to be adapted to the behavioral needs. This means to refine situation models to reduce uncertainty in the expected outcome of this action. We call this process semantic knowledge generation as it allows to explain and eliminate differences between simulated and real world by refining the situation models through structural learning mechanisms like specialization. This leads to a refined situation space in which a specialized behavior depending on the feedback is possible.
  Depending on the agent’s experience, the structural refinement might not always be optimal and could produce a hierarchical deep redundancy in the situation space. To prevent this the mechanism of generalization is used. This mechanism is triggered by the graph motif explained earlier and reduces unnecessarily specialized situations. The target of the interplay between specialization and generalization is the convergence toward a situation space that is minimal (in terms of situation numbers and hierarchical levels) but still optimal for behavioral performance.
- Behavioral Learning
  Determines the optimal action for the new refined situation model. In our current implementation, semantic knowledge generation enables the agent to react with a refined behavior to target or avoid certain types of feedback. Thus, maximizing the expected feedback is the main target of this learning unit. Reinforcement learning methods are predestined to solve such optimization tasks. Here, we have used Q-learning to determine the optimal action. At this point of research, each behavioral model ${ bm}_i$, thus each level of the hierarchy, contains it’s own decoupled Q-learner $Q_i:S_i \times A_i \rightarrow R$, where $S_i$ and $A_i$ is a set of situation and actions of ${ bm}_i$. In future work, this may be replaced by a fully hierarchical Q-learner like MAXQ [42].

Semantic knowledge generation and behavioral learning closely interact with each other. By generating new semantic knowledge a behavioral optimization is possible. In turn, once behavior has changed, it may be necessary to adapt the world model again, for example, as the actions performed by the optimized policy may have unpredictable outcomes again.

To control this cooperation of semantic knowledge generation and behavior learning is a difficulty that is not fully solved at this point. After a situation is refined by semantic knowledge generation, the optimal situation–action mapping has to be determined through reinforcement learning. While behavioral learning is in progress semantic knowledge generation is deactivated. Once the behavioral learning has converged, the semantic knowledge generation can be activated again.

#### Structural Learning

As described above, we use feedback $f=(l,r)$ as a measure of expectation. Thus, a difference between expected feedback and actually gathered feedback leads to structural refinement by specialization. By doing so, the implemented mechanisms of knowledge refinement are special cases of the basic mechanism explained in the previous sections, where generally every unexpected part of a situation contributes to this measure. In our current implementation of the specialization mechanism, we differentiate between “gathering feedback when expecting feedback, but gathering the wrong type/value of feedback” and “gathering feedback when not expecting any feedback.” The first case triggers the process of feedback specialization and the second one triggers feedback expectation. Both cases are special cases of the general graph motif for specialization.

Specialization enables refined behavioral learning. Thus, for every new situation the optimal action can be determined as explained earlier. To learn the optimal policy, already experienced memory can be used to “pre-learn from mind” (use knowledge from the episodic memory to perform offline learning). This allows the agent to perform a more accurate educated guess for the optimal action in the next learning cycle.

**Fig. 10:**
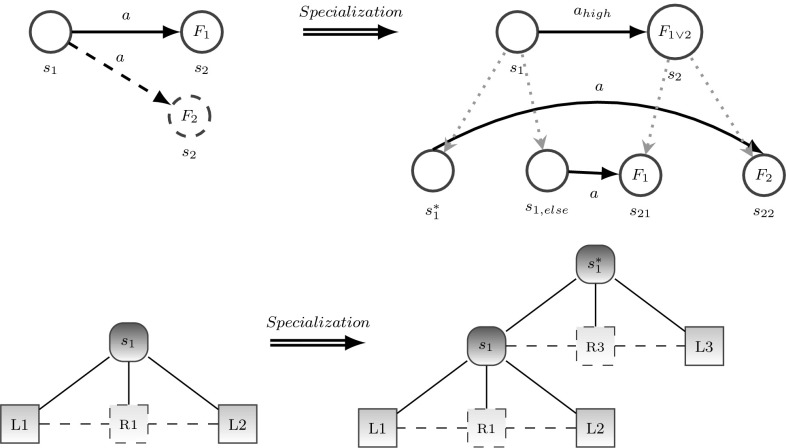
Feedback specialization refines the behavioral model as well as the scene entity model to improve the behavior

*Feedback specialization* is triggered if some feedback was expected in a situation, but a different feedback occurred. Thus to enable the agent to react specifically to a certain outcome (feedback) of an action starting from the same start situation, this situation has to be specialized into two situations. As shown in Fig. 10, executing action *a* in situation *s* usually leads to the situation *s*1 with the expected feedback $F_1$ but sometimes the unexpected feedback $F_{2}$ is gathered. This is a trigger for feedback specialization. This means to extend the behavioral model, especially replacing action *a* by a high-level action $a_{{high}}$ by specializing the start situation $s_1$ into two new situations $s_1^*$ and $s_{1,{else}}$ such that performing action *a* in situation $s_{1,{else}}$ leads to the previously expected feedback $F_1$, whereas choosing *a* in situation $s_1^*$ leads to the new previously unexpected feedback $F_2$. The scene entity model of $s_1^*$ is a specialization of $s_1$ and extends $s_1$ by some knowledge as illustrated in 10 (bottom). There are now two goal situations of $a_{{high}}$, $s_{21}$ and $s_{22}$ with the same scene entity model as $s_2$, but one expecting the feedback $F_1$ and one expecting $F_2$. The sensory configuration of $s_2$ is not illustrated, due to the fact that the sensory configuration does not change.

*Feedback expectation* consists of two parts. The first one is feedback interpretation and the second one is feedback prediction. This process enables the system to react to any feedback at any time. Once a feedback is received, when no feedback was expected, the agent tries to explain why feedback occurred, to expect and predict it in the future. This method also enables the agent to split one high-level action into several sub-actions (chaining) and enables the specialization of subtasks.

**Fig. 11:**
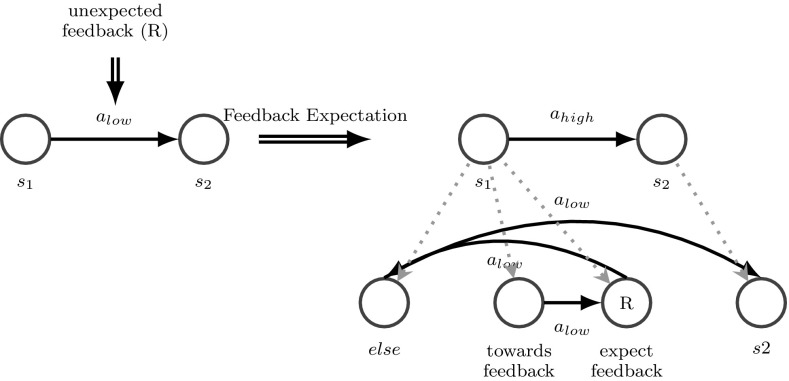
Feedback expectation and expectation specialization are both used to explain and predict external feedback. If no feedback is expected, but feedback occurs, feedback expectation is performed. If feedback is expected, but a different feedback occurs expectation specialization is performed

As shown in Fig. 11, once the agent receives feedback, the agent tries to explain when this feedback occurs (feedback situation) during this event. In a next step, the agent checks the near past before this feedback and tries to create some predictor (toward the feedback situation). In the current implementation, the predictor evaluates the structure of the raw sensory scene representation, in particular, those objects that have been selected to explain the feedback, and trains a different classifier with the attributes of the objects leading to the feedback. It has to be mentioned, that this is a restriction, because the feedback has to be predictable by the same objects which are causing the feedback.

If different types of feedback occur before the agent is able to finalize the expectation task, due to the lack of information gathered to explain one feedback, the information might be enough to predict the general event of feedback. In further steps, this prediction of general feedback can be specialized to predict either of the feedbacks. The information that there will be feedback might be very helpful for realizing attention control, for example, by focusing processing resources specifically on the difference between the feedbacks. However, this aspect is not further targeted at this stage of research.

#### Semantic Knowledge Generation

An important part of self-referential learning is the generation of important knowledge from experience. Semantic knowledge is important to understand a situation and create a model of the environment based on behavioral needs. The reason to extract knowledge is because the internal world model differs from the external input. Thus, the internal world model has to be refined.

**Fig. 12:**
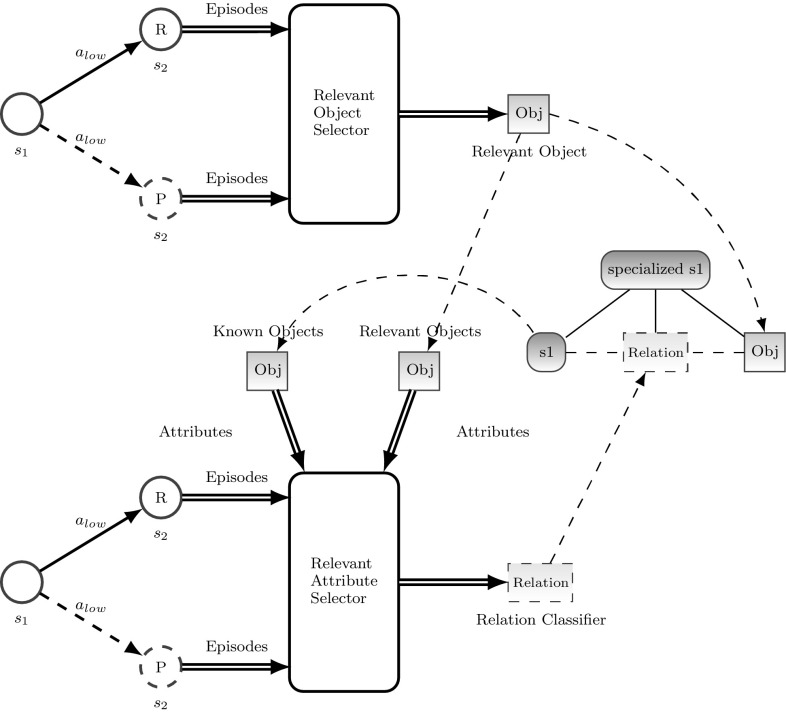
Semantic knowledge generation. The first stage extracts the most relevant objects to explain the difference in expected and real feedback. The second stage determines all relevant attributes of new and already-known objects to train a relation classifier. Both, new relevant objects and relations are used to refine the situation

In our current implementation, we use a two stage knowledge extractor as shown in Fig. 12. The first stage is fed with the clustered set of episodes, which belong to the actual event. The clustering separates the episodes which can be explained by the internal model from those which cannot be explained. Based on these clusters, a feature selector calculates the information gain for each involved object to measure the object’s relevance for discriminating between these different clusters (cf., [43]). If the information gain is above some threshold, the type of object is considered as “relevant for the situation“. This object represents a leaf node in the sensory configuration.

The second stage takes all relevant objects, which includes the already-known relevant objects and the new gathered relevant objects and performs the information gain calculation on all attributes of these objects. Again if the information gain is above some threshold the attribute is considered as relevant.

Finally, based on the relevant attributes, a classifier with dimension of relevant attributes and based on the clustered episodes is trained. This classifier represents a *relation* in the sensory configuration. Based on the new relation classifiers and the new relevant objects, the internal world model can be refined. The following gives a more detailed description of the algorithm:

1. from the episodic memory collect episodes ${ ep}$ with the same start situation $s_{{start}}$ and the same action *a*
2. cluster these episodes by the classes $C_{{exp}}$ and $C_{{unexp}}$. $C_{{exp}}$ defines the class of episodes that lead to the expected end situation $s_{{end},{exp}}$. $C_{{unexp}}$ defines the class of episodes that lead to an unexpected end situation $s_{end,unexp}$.
3. determine relevant objects *relObj*
  - loop through entire situations *es* belonging to all episodes *ep* inside the classes $C_{exp}$ and $C_{unexp}$ extract all appeared object categories $O.A('{} { category}')$ into ${ CAT}_{all}$.
  - loop through entire situations ${ es}$ belonging to all episodes ${ ep}$ inside the classes $C_{exp}$ and $C_{unexp}$ and determine the appearance value $app_{v}$ for every category in ${ CAT}_{all}$, which is ${ True}$ is an object of that category appears in the entire situation and ${ False}$ if not.
  - for each category ${ cat}$ in ${ CAT}_{all}$ calculate the information gain *I* based on the classification of the two categories $C_{exp}$ and $C_{unexp}$.
  - relevant objects ${ relObj}$ are objects of a category with an information gain above some threshold.
4. determine the relation for the refined situation
  - for both classes $C_{exp}$ and $C_{unexp}$ collect all entire situations that contain all relevant ${ relObj}$ and all known objects ${ knownObj}$. Known objects are objects, that are already part of the scene entity model of the situation $s_{start}$.
  - for every attribute of every known and relevant object calculate the information gain. The attributes with an information gain above some threshold are defined as relevant attributes and form the feature space of a relation classifier introduced earlier.
  - train a new relation classifier ${ relClassifier}$ based on the two classes $C_{exp}$ and $C_{unexp}$.
5. integrate the new relevant objects ${ relObj}$ as well as the relation classifier ${ relClassifier}$ into the scene entity model as shown in Fig. 12.

The node ${ specialized s1}$ defines the new specialized situation of the start situation *s*1 and allows now the refinement of the behavioral model and a refined behavior.

## Simulation Experiments

### Scenario

**Fig. 13:**
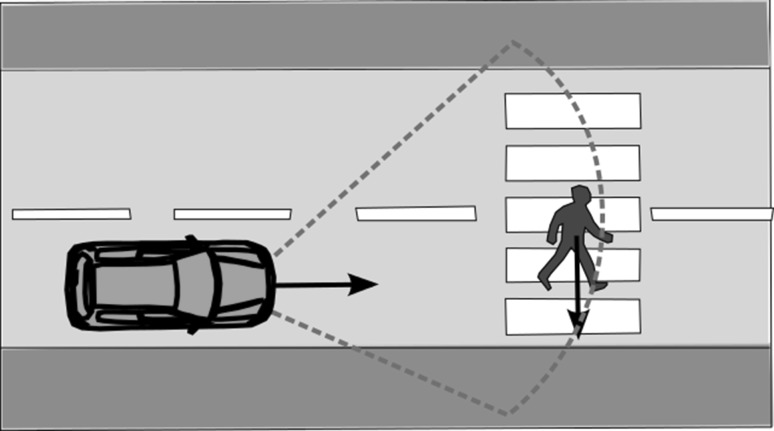
Scenario

The purpose of the following scenario is to demonstrate aspects of self-referential autonomous learning and to show the feasibility of our approach. This means to learn new situation and behavioral models in a simple simulated traffic scenario. Here, an agent has to learn to drive a car safely over a zebra crossing as illustrated by Fig. 13: More specifically, the scenario is that of a car approaching and driving safely over a zebra crossing without intimidating or hurting pedestrians crossing the street, but also without unnecessarily wasting time. The system will be provided initially with some mid-level preprocessed sensory information such as ego-velocity, location of zebra crossing, as well as probable locations of pedestrians. Thus, in our scene representation there are several types of objects possible which are listed in Table 1 with their attributes. As our current implementation has only a “symbolic” interface with the CarD traffic simulation environment, the scene ontology of objects is assumed to be fixed in the following, and structural learning is limited to the situation and behavior models for driving over the zebra crossing.

**Table 1:** Types of objects as used for scene representation

Pedestrian object	House object	AgentCar object	Wheelchair object
Category = pedestrian	Category = house	Category = self	Category = wheelchair
xpos	xpos	xpos	xpos
ypos	ypos	ypos	ypos
Orientation	Orientation	Orientation	Orientation
Velocity	Color	Velocity	Velocity
Age		Lights on	

Additionally, the system is provided with a high-level behavioral model (that could be communicated, for example, by a driving teacher) in order to specify the task and the high-level processing steps to solve the task. This boils down to the specification of a set of rules such as “IF not arrived at target position THEN continue driving.” Of course such models are incomplete as they lack (1) situations not preconsidered by the teacher, (2) full specification of situation models and (3) full specification of action models and their integration with lower-level behavioral models referred to in the teacher model. In order to complete learning of full behavioral and situation models, the system has to (1) explore the state space, (2) integrate low and high-level behavior models , (3) learn new situation models (at intermediary levels) as required by the task, and (4) optimize behavior, e.g., with respect to time and/or energy costs. Here, the costs correspond to either the reward obtained for arriving at the target location by driving safely over the zebra crossing or the punishment for hurting pedestrians. This general procedure can be pursued in scenarios of various difficulty degrees as explained below. We assume high-level actions “stop” for stopping in front of a stop line and “drive” for accelerating to a certain speed and then keeping this speed.

### Scenario1: Learn to Understand the Meaning of a Pedestrian and Wheelchair for Driving Safely over a Zebra Crossing

**Fig. 14:**
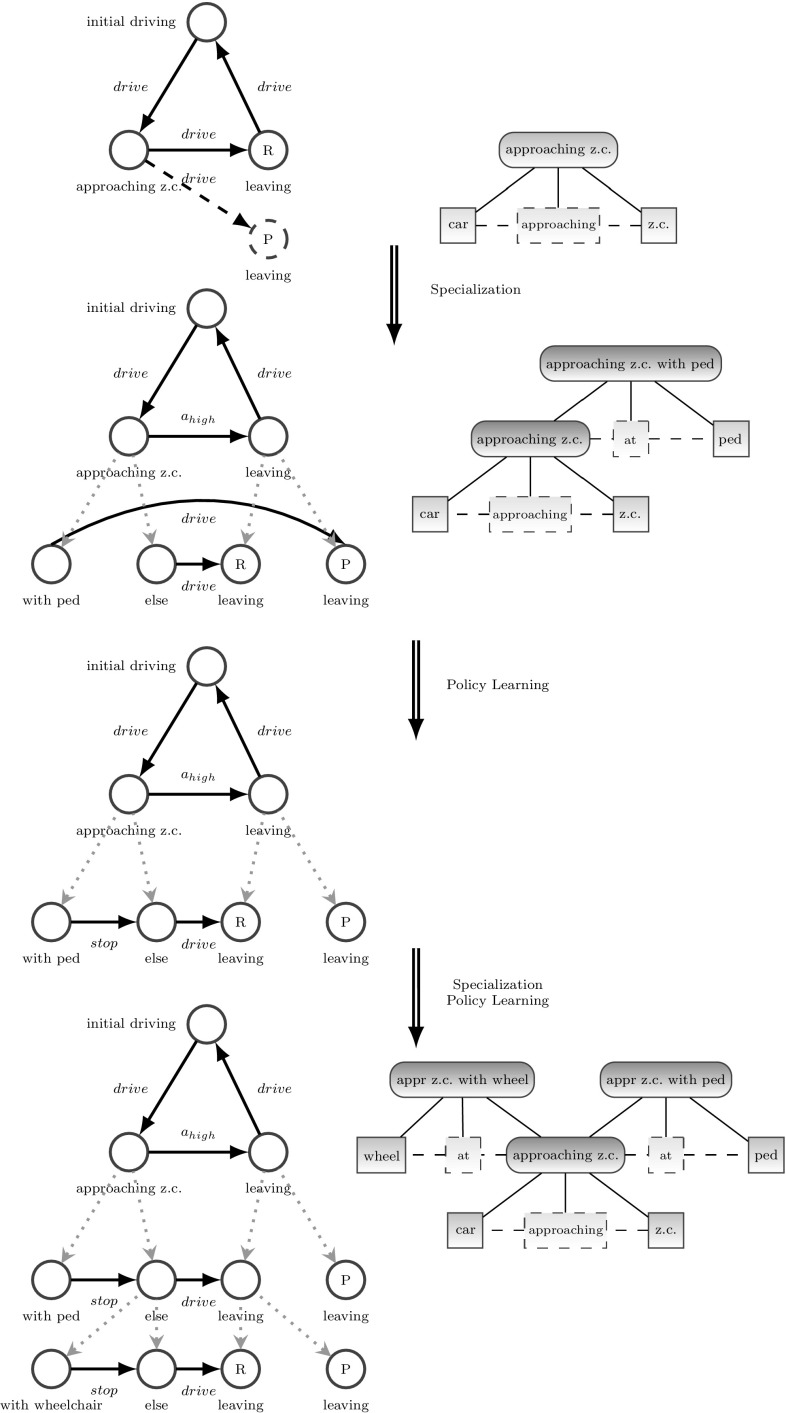
Scenario: specialization and Policy Learning starting from teacher-given behavioral model (*top*, *left*) and scene entity model for approaching z.c. (*top*, *right*) in 4 steps down to the final learned behavioral model (*bottom*, *left*) with the extended sensory configuration (bottom, right). The steps are Specialization to understand the meaning of pedestrians at a z.c. followed by policy learning to obtain the best action in the new situation, followed by specialization to understand the meaning of a wheelchair at a z.c. again with policy learning

We introduce a scenario with a teacher model for driving from a start location to an end location. Here, the teacher model, illustrated in Fig. 14 (top), is essentially a behavioral model with three situations, “driving free,” “approaching z.c.,” and “leaving z.c.” In all three situations, the action ”drive“ is selected by the teacher to be performed. “driving free” is active as long as none of the other situations is active. “approaching z.c.” becomes active as soon as the agent car is within a bounding box around the zebra crossing and approaching the zebra crossing. “leaving z.c.” gets active for the moment the agent leaves the z.c. This situation expects some reward for successfully driving over the zebra crossing.

By performing the teacher model, the system will initially be able to perform the task quite well as long as there are no pedestrians or wheelchairs crossing the z.c. However, once hurting or intimidating a pedestrian or a wheelchair user, the agent will experience punishment. Thus, the initial model is not optimal and fine enough to perform the task sufficiently (meaning not hurting anybody while crossing the z.c.).

#### Results

**Fig. 15:**
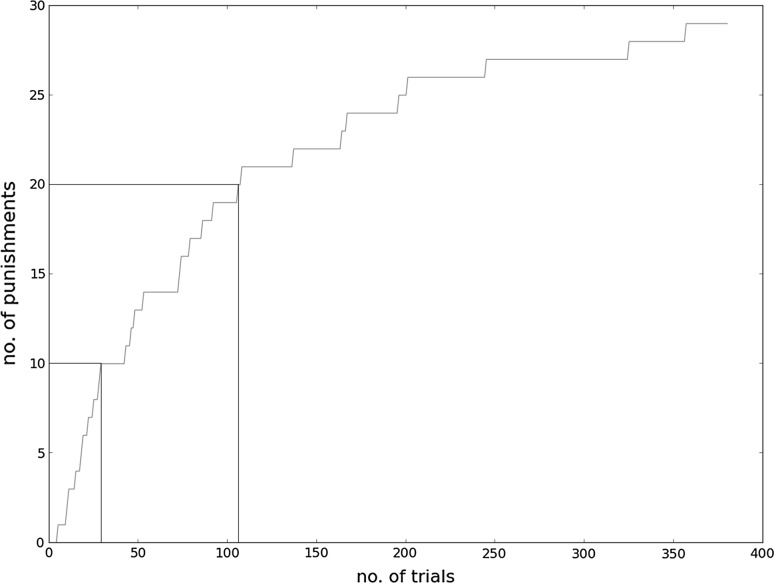
Cumulative number of punishments. 1st phase ($0<t<30$): behavior defined by teacher model; 2nd phase ($30<t<110$): refined model after learning to cope with a pedestrian at a z.c.; 3rd phase ($t>110$): refined model to cope with both pedestrians and wheelchairs at a z.c.

After a preset number of occurrences of the motif for feedback specialization (expecting some feedback, but archiving a different feedback), this mechanism gets active. As seen in Fig. 14 (from top to bottom), the agent first determines the occurrence of the pedestrian in a certain relation to the z.c. and the agent car as the cause for the unexpected feedback. This induces feedback specialization of the situation “approaching z.c.” into “approaching z.c. with ped” and “else.” Based on the refined behavioral model, Q-learning is used to determine the optimal action for each of the new situations, with the result, that the agent performs the action “stop” in “approaching z.c. with ped” and “drive” in “else.” The “else“ situation represents basically the situation “approaching z.c. without ped.” As shown in Fig. 15, the value of punishments/tries is significantly reduced after this step. But there are still punishments left. This is due to the occurrence of wheelchairs which cross from time to time and are not detected as pedestrians. Thus, after collecting some more punishments, our system determines the occurrence of the wheelchair in a certain relation to the z.c. and the agent car as the cause for the unexpected feedback (punishment), and, as before, the behavioral model is extended by the situations “approaching z.c. with wheelchair” and “else.” After determining the optimal actions, the value of punishments/tries significantly decreases again.

Thus, after self-referential learning, our system is able to understand the meaning of a pedestrian and a wheelchair crossing a z.c. and also to perform the optimal action in each of the situations.

### Scenario2: Learn to Understand the Meaning of a Zebra Crossing in Combination with a Crossing Pedestrian for Driving Safely Along a Road.

**Fig. 16:**
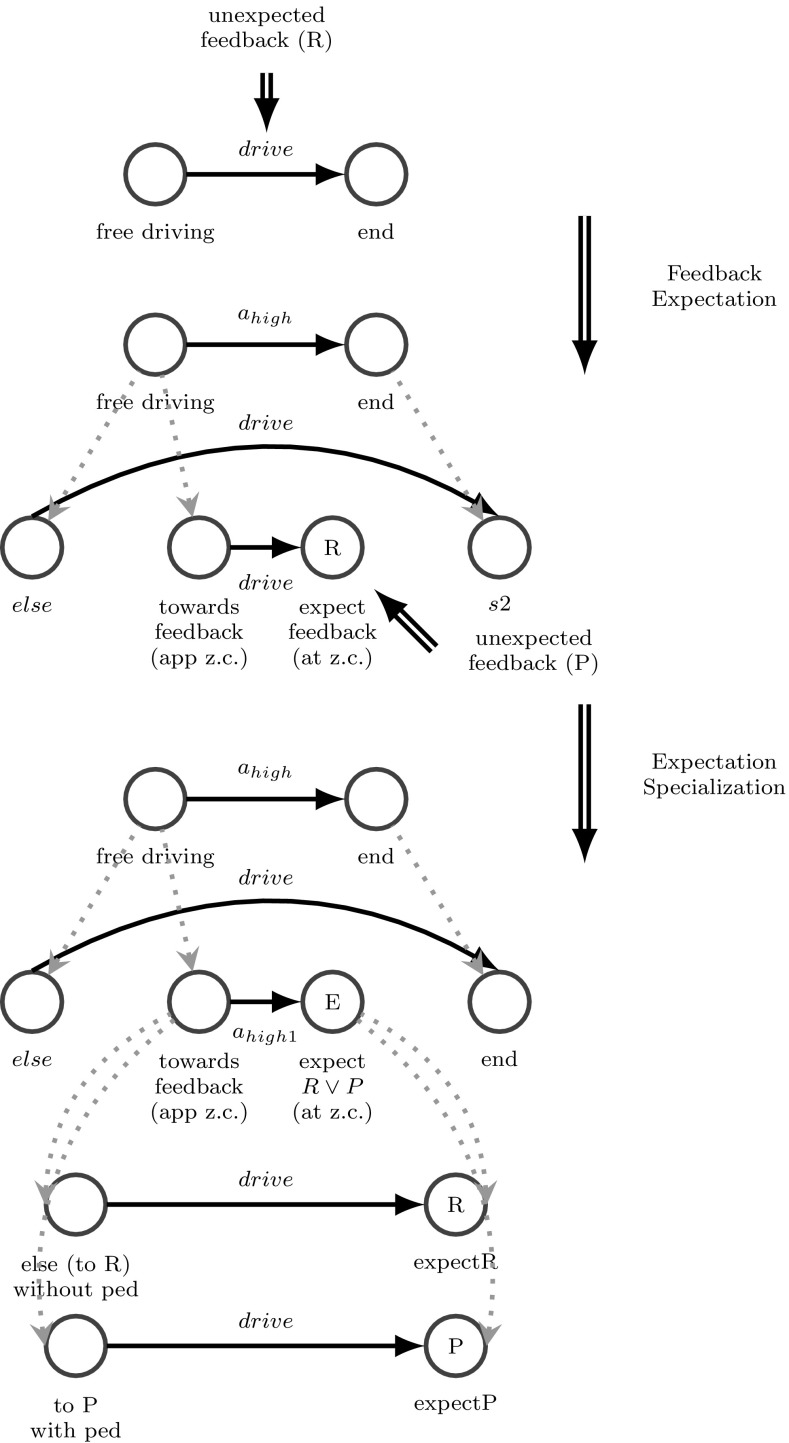
Feedback expectation applied to scenario 1 to explain and predict the feedback when driving over a zebra crossing (reward for crossing without getting too close to a pedestrian and punishment for getting so close to a pedestrian while crossing)

We introduce a scenario with a teacher model for driving from a start location to an end location. Here, the teacher model, illustrated in Fig. 16, is essentially a behavioral model with two situations, “driving free” and “end.” In “driving free,” the action “drive” is selected by the teacher to be performed. “driving free” is active as long as the agent didn’t arrive at the end location. Compared to the previous scenario, there is no knowledge about a zebra crossing and no situation that determines when the agent is approaching a z.c. Also, no feedback is expected by the agent during the task. By this, the system will initially be able to perform the task quite well as long as there is no z.c. with pedestrians. Once the z.c. is crossed some kind of feedback is archived by the agent, which is not expected: usually more often the reward signal for crossing safely, but sometimes as well punishment for crossing and hurting pedestrians. The very limited initial model is not optimal and fine enough to perform the task sufficiently (meaning not hurting anybody while driving). Thus, neither the meaning of “zebra crossing” nor a corresponding behavioral model for such situations is pre-designed by the teacher.

#### Results

After a certain number of occurrences of the motif for feedback expectation (expecting no feedback, but archiving some feedback), this mechanism gets active. As seen in Fig. 16, the agent first determines the occurrence of the z.c. in a certain relation to the agent car as the cause for the unexpected feedback. Thus, based on the feedback expectation mechanism the situation “driving free“ is specialized to two situations: “feedback at z.c.” is expecting the feedback right when crossing the z.c. “approaching z.c.“ is the predictor for the feedback. Thus, based on all entire situations right before the actual feedback, which contain the relevant objects z.c. and the agent car, this predictor is trained. The resulting refined behavioral model is shown in Fig. 16 (middle). At this point the situation, “feedback at z.c.” is expecting some reward, because this happened much more often than punishment for hurting pedestrians. Thus, the agent is able to predict and understand the meaning of a zebra crossing, but not yet the meaning of pedestrians at a zebra crossing. The result of this scenario may be the basis for Scenario 1, which uses feedback specialization together with behavioral learning (RL) to refine the model further and optimize behavior as shown in Fig. 16 (bottom).

## Summary and Discussion

Scene understanding and situation recognition is an essential technological prerequisite for intelligent systems applications such as autonomous driving or mobile human support systems [44, 45]. Current approaches toward scene analysis and situation understanding face several essential problems. For example, real-world scenes are too complex and undergo too many variations that simple appearance-based methods would be useful for predicting future episodes of behavior in similar situations [10–12]. Moreover, state-of-the-art systems cannot adequately solve the problem of autonomous learning of structured scene or situation representations that are usefully constrained by behavioral needs [17, 29, 32]

In this work, we have developed a cognitive architecture for self-referential autonomous learning of situation representation. Our system is inspired by brain architecture based on neuronal cell assemblies and associative learning [46–50], and comprises subsystems for working memory, episodic memory, and semantic memory including structured situation models and hierarchical behavioral models for planning and decision making [13, 29, 32, 51]. By self-referential learning, we mean the control process of autonomously extending subjective knowledge representations. Similar to hierarchical reinforcement learning (HRL), this involves behavioral optimization of hierarchical policy models [29, 32, 42, 52–56]. There, so-called options $({\mathcal {I}},\pi \beta )$ are used for structuring the action and situation space, where a policy $\pi$ can be activated if the agent is in a primitive state $s\in {\mathcal {I}}$ that belongs to the set of the option’s initial states ${\mathcal {I}}$ and will be followed until the agent’s state is in some target set $s\in \beta$. Thus, *s* and ${\mathcal {I}}$ correspond to different levels of a hierarchical situation model, and HRL provides methods to optimize option policies. While most HRL approaches assume a given pre-designed hierarchical structure [32, 36, 42, 57] or only bottom-up learning from the level of primitive states [53, 54, 58], our approach targets at general structural learning of behavioral and situation models by extending “is-a” and “has-parts” ontologies of situation models, including both specialization and generalization [16–18, 40].

Besides proposing basic architectural concepts, we have also described a first implementation of our architecture. This implementation was tested within a simple simulated traffic scenario to demonstrate the viability of our approach. Instead of unconstrained bottom-up construction of novel situation and behavior models, our system adopts an abstract model that is communicated by a teacher in terms of the current knowledge representations. By trying to execute the abstract teacher model, our system can produce structured episodic data and evaluate the outcome through a reward system. Triggered by the mismatch between predicted and actual action outcome, our system can exploit the episodic data for structural learning. This results in a hierarchical extensions of situation models and their integration into a refined behavioral model. Therefore, our system is able to autonomously learn novel situation types and integrate them into the ontology of previously acquired knowledge.

Although the current implementation solves only quite a simple problem, we think that our approach scales favorably to more complex tasks. For example, one potential problem is the proliferation of situation nodes in the behavioral models that may occur in bottom-up approaches due to uncontrolled specialization, in particular, in stochastic environments including partially observable or hidden states [31, 39]. As our system includes both specialization and generalization as well as a way for a teacher to communicate high-level abstract models to specify a raw solution to a task, this will strongly constrain the agent’s search space and keep the clustering procedure described in “Semantic Knowledge Generation” section feasible.

Still, our learning algorithm may be too complex for an immediate online implementation of autonomous learning in current embedded automotive hardware. Instead, we rather consider the support of model design as potential short-term applications of our system: This can be achieved, for example, by simulating complex traffic scenes to let the agent learn an adequate structure for the situation and behavioral models that may later be employed in real vehicles. In addition to simulations, the “self-referential” loop (Fig. 7) may be closed by collecting episodic traces recorded from real driving vehicles, whereas the structural model updates would occur offline. On the long term, however, we believe that full online autonomous learning cannot be realized much cheaper than in our system. In future work, we therefore will extend our system toward more complex application scenarios and additional types of self-referential structural learning.
